# Phenotypic selection on ponderosa pine seed and seedling traits in the field under three experimentally manipulated drought treatments

**DOI:** 10.1111/eva.12685

**Published:** 2018-12-19

**Authors:** Marcus V. Warwell, Ruth G. Shaw

**Affiliations:** ^1^ U.S. Department of Agriculture, Forest Service Rocky Mountain Research Station Moscow Idaho; ^2^ Department of Ecology, Evolution, and Behavior College of Biological Sciences University of Minnesota‐Twin Cities Saint Paul Minnesota

**Keywords:** adaptive variation, aster models, date of emergence, drought selection, growth rhythm, natural selection, *Pinus ponderosa*, unconditional expected height

## Abstract

Drought‐related selection during seedling emergence and early development may play a strong role in adaptation. Yet this process is poorly understood and particularly so in relation to ongoing climate change. To evaluate drought‐induced differences in selection during early life stages, a total of 50 maternal families sampled from three climatically disparate ponderosa pine (*Pinus ponderosa* Doug.) populations were grown from seed in two common garden field experiments at a location that was warmer and drier than seed origins. Three drought treatments were imposed experimentally. Phenotypic selection was assessed by relating plant fitness measured as survival or unconditional expected height at age 3 to seed density (mass per unit volume), date of emergence, and timing of shoot elongation. In the year of emergence from seed, differential mortality was particularly strong and clearly indicated selection. In contrast, selection in subsequent years was far less pronounced. Phenotypes with high seed density, an intermediate but relatively early emergence date, and high 2nd‐year early‐season shoot elongation exhibited the greatest estimated fitness under drought. The form of selection varied among seed sources in relation to drought treatment. Selection was generally more acute in the cases of greatest difference between drought treatment and climatic patterns of precipitation at the site of seed origin. These results suggest that populations of ponderosa pine are differentially adapted to drought patterns associated with the climate of their origin. To the extent that the phenotypic traits examined are heritable or correlated with heritable traits, our results provide insight into how tree populations may evolve in response to drought.

## 1 INTRODUCTION

Forest tree populations produce immense numbers of genetically variable seeds (e.g., ~93,076 seed per ha in a year, Greene & Johnson, 1994) which are commonly subject to high drought‐related mortality during emergence and seedling development (Haig, Davis, & Weidman, 1941; Moles & Westoby, 2004). In addition, emerging and early juvenile stages are more susceptible to climate‐related mortality than later life stages (e.g., Haig et al., 1941; Leck, Parker, & Simpson, 2008). Therefore, although mortality is not necessarily selective, drought‐related mortality has the potential to act as a strong selective filter during early life stages, and it may, in turn, have a substantial effect on a population's genetic constitution, adaptation, and performance during later life stages.

The analysis of natural selection on phenotypic variation represents a first step in predicting evolution of quantitative traits in response to selection. Phenotypic selection studies generally detect selection on traits and describe the form and magnitude of selection (see Kingsolver & Pfennig, 2007). Evolutionary response is expected when a trait under selection exhibits heritable variation within a population (Darwin, 1859; Endler, 1986; Falconer & Mackay, 1996), and short‐term evolutionary response to selection can be predicted when estimates of selection on the phenotype and trait heritability are available (Falconer & Mackay, 1996). Study results may also be used to gain insight into possible mechanisms of selection (Mitchell‐Olds & Shaw, 1987).

Studies of trees have provided considerable evidence that populations possess potential to adapt to changes in drought conditions. For example, intrapopulation genetic variation for traits associated with growth and survival in response to variation in moisture availability has been observed in seed weight, color (e.g., Ager & Stettler, 1983; Van Deusen & Beagle, 1970; Weber, 1988), date of germination or emergence (e.g., Jenkinson, 1977; Feret, 1982; *Pseudotsuga menziesii*, Bai, Thompson, & Broersma, 2000), tap root length (e.g., Jenkinson, 1975; Feret, 1982; *Adansonia digitate*, Cuni Sanchez et al., 2011), and root/shoot biomass ratios (e.g., Feret, 1982; McMillin & Wagner, 1995; *A. digitate*, Cuni Sanchez et al., 2011).

There is also substantial evidence that some tree species have adapted to variation in moisture availability across their distribution, which suggests that drought‐related selection has operated on trait variation within tree species. For example, seeds collected from increasingly drier origins across apparent moisture gradients exhibit higher seed weight (e.g., Ager & Stettler, 1983), seed mass (e.g., *Quercus suber*, Ramirez‐Valiente et al., 2009), relative germination speed (e.g., Weber & Sorensen, 1992), and germination percentage under moisture stress (Moore & Kidd, 1982). In common garden studies, more xeric seed sources exhibited increased growth rates (*Q. oleoides*, Ramirez‐Valiente et al., 2017), increased intermittent (lammas) shoot growth (e.g., *Pseudotsuga menziesii*, Kaya, Adams, & Campbell, 1994), and allocated a greater proportion of their biomass to roots (e.g., *P. sylvestris*, Matías, González‐Díaz, & Jump, 2014). Notably, among 138 *P. ponderosa* provenances, height growth of three‐year‐old trees was greatest for seed originating from drier locations on a xeric common garden site, while on a mesic common garden site, trees representing genetic sources originating from wetter locations were tallest (Rehfeldt, 1986).

However, basic knowledge of what range of quantitative traits are selected for by natural selection under drought stress and how this selection may vary within and among populations of forest trees is not well developed (see Alberto et al., 2013). In particular, knowledge of the magnitude and direction of natural selection on emergence and early juvenile traits under drought conditions is very limited. In the case of forest tree species, this consideration is particularly relevant to western North America, which is expected to be subjected to warmer and drier climates (IPCC, 2013) and greater temporal frequency and spatial extent of growing‐season drought events through the 21st century (Sheffield & Wood, 2008).

**Figure 1:**
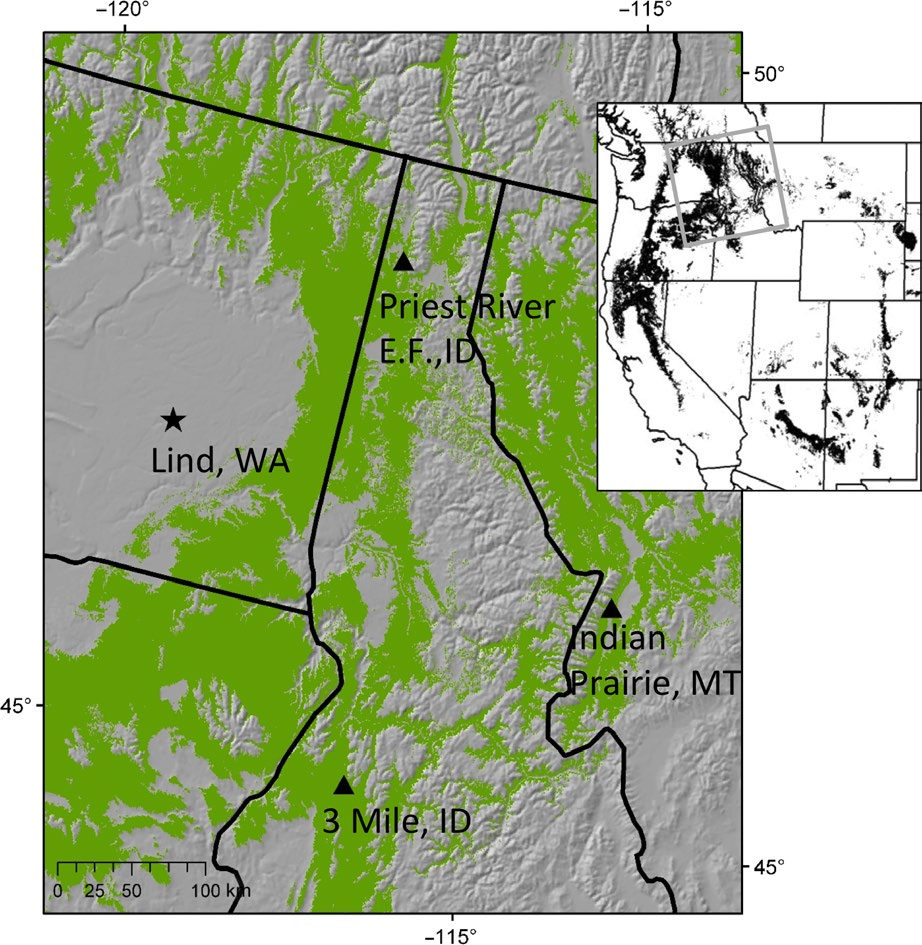
Location of provenances (▲) and study site (★). Green indicates predicted realized climate niche of ponderosa pine (*Pinus ponderosa*) (Rehfeldt, Crookston, Warwell, & Evans, 2006). Upper right inset shows western contiguous United States with dark regions indicating the range‐wide distribution of ponderosa pine (Little, 1971) with the southern range updated to reflect recent taxonomic reclassifications (Perry, 1991). The area represented in the larger image is indicated by the gray rectangle

Therefore, as a key step in an investigation of how forest tree populations may evolve under ongoing climate change, this study assessed natural selection in ponderosa pine (*Pinus ponderosa* Doug.) during early life stages following emergence in the field under climate warmer and drier than seed origins and under three experimentally imposed drought treatments. Ponderosa pine is of particular interest given its status as both an economically and ecologically important tree species in western United States (Van Hooser & Keegan, 1992). The species naturally occurs in both warm‐moist and warm‐dry climate (Rehfeldt, Ferguson, & Crookston, 2008) across a broad geographic range that spans much of western United States and southern British Columbia (Figure 1, inset) (Little, 1971; Perry, 1991). Throughout its distribution, ponderosa pine defines the lower tree line and represents the warm‐dry limits of the coniferous forest and is among the most drought‐tolerant tree species native to North America (Steele, 1992). Nonetheless, drought poses a significant barrier to early seedling establishment in ponderosa pine (Curtis & Lynch, 1957; Foiles & Curtis, 1965) and may act as a primary selection pressure throughout its distribution (Weber, 1988).

This study addressed the question: (1) What are the magnitude, form, and temporal dynamics of selection on seed and seedling traits under different regimes of the timing of growing‐season drought for (a) a single provenance and (b) among three climatically disparate provenances from the Rocky Mountains in the Northwestern USA. Given the evidence from adaptive patterns presented above, we hypothesized that with increasingly earlier growing‐season drought, directional selection will increasingly favor traits that contribute to early resource acquisition and drought tolerance such as increased seed size, earlier seed emergence, and higher early‐season growth rates. Furthermore, we hypothesized that seed source originating from increasingly drier climate will exhibit less selection in response to increasingly earlier growing‐season drought.

## 2 MATERIALS AND METHODS

### 2.1 Planting site

To evaluate phenotypic selection of ponderosa pine during early seedling establishment under warm‐dry climate and experimental watering treatments, two common garden experiments were established at the Washington State University–Dryland Experimental Station in Lind, Washington (47°00′12′′N, 118°33′46′′W, elev. 491 m.a.s.l.). The study site was considerably warmer with substantially lower precipitation than climate of the sampled population origins (Table 1). Its Ritzville silt loam soil is characteristically well drained (Whitney, 1916). Otherwise, its microhabitat characteristics (i.e., aspect, solar radiation dynamics, and wind shelter) were well suited to ponderosa pine regeneration as described by Munger (1917) and Heidmann (1992).

**Table 1:** Population origins and their predicted climatic means (Rehfeldt, 2006) and the study sites^a^ location and its observed mean climate for 2008–2011 (WSU‐DRS 2015)

	Longitude	Latitude	Elevation (m)	Mean annual temperature (°C)	Mean annual precipitation (mm)	Growing‐season precipitation (April–October)
3 Mile, ID	116.22°W	44.98°N	1,341	5.4	661	239
Indian Prairie, MT	114.17°W	46.47°N	1,219	6.1	428	217
Priest River Exp. For., ID	116.82°W	48.35°N	808	6.4	819	287
Lind Dryland Exp. Sta., WA^a^	118.57°W	47°N	501	9.4	251	86

### 2.2 Experiment 1

Experiment 1 (Exp. 1) was designed to reveal variation in phenotypic selection among maternal families within a single population. Seeds were from wind‐pollinated cones, collected in fall 1989 from 36 trees with crown positions that were either dominant or codominant relative to the forest canopy in five natural stands at Priest River Experimental Forest, Idaho (Figure 1). Sampled trees were located within a two‐mile (~3.2 km) radius of each other at elevations between 800 and 950 m.a.s.l. and represent a single altitudinal provenance or seed zone (see Rehfeldt, 1991). The same seed collection was used by Marshall, Rehfeldt, and Monserud (2001) to assess family differences among seedlings for height growth and photosynthetic traits in common gardens in their local environment. This seed collection was also from the same maternal trees used by Marshall and Monserud (1996) for tree ring analysis.

### 2.3 Experiment 2

Experiment 2 (Exp. 2) was designed to assess variation in phenotypic selection among populations. Seeds were from wind‐pollinated cones, collected in 1982 from eight wild trees from each of three representative localities from the Rocky Mountains in the Northwestern USA. The provenances (geographic origin of seeds) were selected based on adaptive differences in growth and shoot phenology (Rehfeldt, 1991) and disparity with respect to climate and geography of origin (Table 1). The seed sources were from the same collection used by Rehfeldt (1992), who used them in common garden studies to estimate genetic variances and covariances for traits describing growth, growth potential, and shoot elongation.

### 2.4 Design and procedure

Mean density (mass per unit volume; mg/ml) of seeds, which has been shown to affect seed vigor (e.g., Hoy & Gamble, 1985), was estimated for each family using the mean density of 20 seeds in five independent measures. Prior to sowing, seed preparation followed methods described by Wenny and Dumroese (1987). For both common garden experiments, seeds were sown in the same week in the fall of 2008 and allowed to stratify over winter in situ. Seeds were sown in pairs at a uniform depth of 1.5 cm at 1.5 cm spacing within rows and 20 cm between rows. Two seeds were planted per position to increase likelihood of consistent spacing. A total of 17,280 and 7,200 seed were sown in Exp. 1 and Exp. 2, respectively. Prior to the implementation of the mid‐ to late‐summer drought treatment (see below), seedlings were thinned to one per position by removing the easternmost seedling.

Both experiments used a randomized complete block design, with five blocks and three drought treatments randomized within each block. The experiments were interplanted by block and treatment so that for example, Block 1, Treatment 1 in Exp. 1 and Exp. 2 were spatially adjacent (Supporting Information Figure S1). Each maternal family was represented in each treatment within block by two row‐plots of eight seedlings in Exp. 1 and two row‐plots with four seedlings in Exp. 2. Precipitation recorded at the study site through the growing season was very low with warm air temperatures (Table 1) (WSU‐DRS, 2015). Thus, seedlings that did not receive supplemental water during growing‐season months (April–October) were expected to undergo drought conditions, particularly during June to September 2009 when precipitation averaged 0.41 cm per month. Initially, all treatments were watered weekly beginning in early spring 2009 prior to emergence. This procedure was intended to approximate high spring moisture availability, which has been positively associated with natural patterns of concentrated seedling recruitment for ponderosa pine (League & Veblen, 2006). The no drought treatment was watered weekly through the growing season. The mid‐ to late‐summer drought treatment was imposed by eliminating irrigation in the last week of June 2009. Finally, a late‐summer drought treatment was imposed by eliminating irrigation in the last week of July 2009. The timing of these treatments corresponds to contemporary patterns of annual drought stress (i.e., late‐summer drought) that are characteristic of geographic origins of the study populations (e.g., Finklin, 1983). All irrigation was applied uniformly using timed hand applications of well water. Because drought treatments within blocks were closely located, moisture‐proof barriers were placed above‐ and belowground between adjacent treatments during irrigation to maintain integrity of drought treatments. No irrigation was applied following 2009.

Extraneous environmental influences were intensively managed in both tests. Fencing was used to exclude ungulates and burrowing mammals from the small (0.02 ha) study site. In addition, hardware cloth was placed over seed beds from seed to early emergence stage to prevent predation from birds and small rodents. Rodent trapping was required during seedling emergence to prevent foraging, and insecticide (imidacloprid: 0.47%, taufluvalinate: 0.61%, and tebuconazole: 0.65%) was applied shortly after emergence to prevent damage from insects. Weeds were removed weekly by hand.

Data were collected for seedling emergence date every 1 to 3 days after the first observed emergent over 30 days in 2009; measures used to assess shoot growth increment were taken in 2010. Bud and lammas shoot growth and total height were assessed in the fall of each year from 2009 to 2011. Survival was assessed every 1 to 14 days through the first growing season and 1 to 3 weeks during the growing season in 2010 and 2011.

### 2.5 Statistical analysis

**Figure 2:**
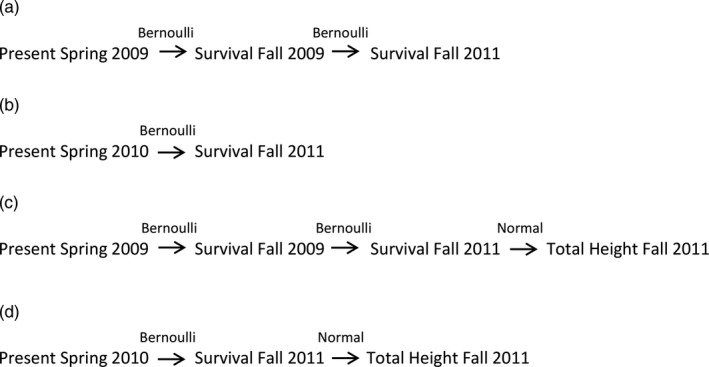
Graphical models for aster analysis jointly analyzing survival over time and total height data to assess fitness. Each node represents a component of life history, while arrows represent the dependent association between predecessor and successor life‐history components. Survival was modeled as a Bernoulli random variable and total height as normal random variable. Graph (a) shows the model used to predict survival in 2011 beginning with seedlings present in Spring 2009. Graph (b) shows model for survival in 2011 beginning with seedlings present in Spring 2010. Graph (c) shows the model used to predict unconditional expected height in Fall 2011 which included survival from Spring 2009 to Fall 2011. Graph (d) shows model for height in Fall 2011 which included survival from Spring 2010 to Fall 2011

To assess natural selection during the juvenile stage, we employed aster models (Geyer, Wagenius, & Shaw, 2007; Shaw et al., 2008; Geyer & Shaw, 2008). The analyses used individual trees as the experimental unit and evaluated the dependence of fitness, comprising survival and final height, on seedling traits, as well as treatment, family, and provenance. They were carried out using mixed‐effects models via the reaster function (Geyer, 2013) implemented in R (R Development Core Team, 2015). Aster models were used, rather than the previously standard multivariate selection approach (Lande & Arnold, 1983), because they permit statistically rigorous, joint analysis of components of fitness expressed during multiple life‐history stages (Geyer & Shaw, 2008; Geyer et al., 2007). The graphical model for fitness as expressed throughout the experiment indicates the dependence of height at age 3 on survival through each year up to that age (Figure 2). Bernoulli distributions were used to model survival following seedling emergence in Spring 2009 to Fall 2011 and Spring 2010 to Fall 2011, and normal distributions were used to model final height in 2011 conditional on survival to that year. In particular, aster was used to estimate unconditional expected final height to characterize overall fitness of plants at age 3. Unconditional expected height differs from the standard measure of height in that it explicitly accounts for the structural zeros due to individual mortality in the estimation of mean height. Therefore, when there is no mortality, unconditional expected mean height is equal to the standard measure of mean height; the estimate decreases with increasing mortality. Subsets of the full graphical model were used in supplementary analyses.

**Figure 3:**
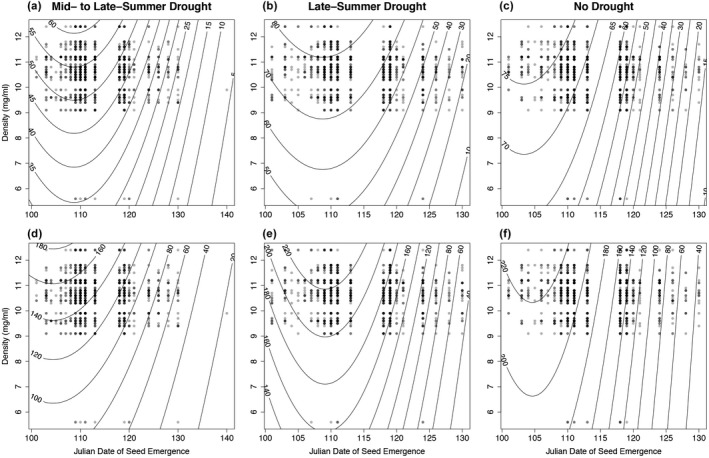
Experiment 1, fitness surfaces showing observed (circles) Julian date of emergence and mean density of seed (mg/ml) by family in relation to modeled (contour lines) survival from 2009 to 2011 (plots a, b, and c) and unconditional expected height (mm) in 2011 that included survival beginning in 2009 (plots d, e, and f) in the mid‐ to late‐summer, late‐summer, and no drought treatment. Increasing darkness of circles indicates more observations for the indicated measures

Analysis of selection on seedling traits and their interactions with drought treatment used the following procedure. First, model comparison using log‐likelihood ratio tests with a forward stepwise regression approach was used to determine the influence of the factors, treatment, family, and provenance on survival and unconditional expected height. Treatment and provenance were treated as fixed, while family, block, and row‐plot were considered random. Then, Spearman's rank correlations between seedling traits were compared for each treatment in each experiment to identify how traits were interrelated and to eliminate serious multicollinearity (r^2^ ≥ 0.70) (see Mitchell‐Olds & Shaw, 1987) in regression analysis. Correlation analysis used only trees that were alive in 2011. Finally, two sets of significance tests were performed. The first set tested whether family mean seed density (mg/ml), and date of emergence were significant predictors of survival from emergence in 2009 to 2011 (Figure 2a) and 2010 to 2011 (Figure 2b) and unconditional expected height in 2011 that included survival beginning in 2009 (Figure 2c) and 2010 (Figure 3d). Results of tests that included and excluded individuals that died in 2009 were compared to deduce how mortality mediated selection in 2009. The second set tested whether family mean seed density (mg/ml), rate of shoot elongation in early season (March 6 to May 7), and mid‐ to late season (May 8–September 28) were significant predictors of unconditional expected height in the 2011 that included survival beginning in 2010 (Figure 2d). To determine direction and magnitude of selection on a given trait requires measures of that trait prior to mortality. Yet shoot elongation could not be assessed until 2010 when it was expressed for the first time. Therefore in this case, the analysis included only those individuals present in 2010. In both sets of analyses, linear selection gradients (*β*_*i*_), quadratic selection gradients (*γ*_*ii*_), and cross‐products (*γ*_*ij*_) for seedling traits were treated as fixed effects while block, row‐plot within block and family were treated as random. Statistical significance of fixed factors was tested by comparing the likelihoods of nested models. Significant (*p* < 0.05) variables were retained. The detection of quadratic terms indicated curvature in the fitness surface. When the quadratic term for more than one trait was significant, the cross‐product for appropriate pairs of traits was included (see Blows & Brooks, 2003). Cross‐product terms (*γ*_*ij*_) represent correlational selection indicating selection on one trait that varies depending on the second trait.

A complete assessment of phenotypic selection using regression analyses requires visualization of the data in relation to the hypothesized model to determine the observed range of traits and to identify potential unexpected patterns or problems in the data (Mitchell‐Olds & Shaw, 1987). Expected fitness (survival and unconditional expected height) was represented using contours while predictor variables (family mean density [mg/ml], emergence date, and timing of the magnitude of shoot elongation rate) were represented on the x and y axes to determine the range of the hypothesized model that was supported by empirical data. The complexity of visualizing shapes in more than three dimensions limits the plots to displaying fitness in relation to two traits.

## 3 RESULTS

### 3.1 Survival and Mean Height

A total of 10,165 seedlings representing 58.8% of the planted seeds emerged in Exp. 1, while 4,564 seedlings representing 63.4% of planted seeds emerged in Exp. 2. Thus, greater seed mortality before emergence may have occurred in Exp. 1. After culling one seedling at positions where two seedlings emerged 7,955 and 3,331 seedlings remained in Exp. 1 and Exp. 2, respectively. These seedlings represented the initial study population. By fall 2011, the initial study population for Exp. 1 and Exp. 2 had undergone 36% and 46% mortality, while survivors exhibited a mean height of 260 mm and 270 mm, respectively. Notably, about 94% of total mortality over the study period in each experiment occurred in 2009 during the growing season in which emergence occurred. Seedling mortality during this period appeared to be associated entirely with drought stress. Dead seedlings were desiccated in appearance and lacked signs of herbivory or physical damage. This conclusion was further evidenced by a positive correlation between mean daily evapotranspiration and timing of mortality; mean evapotranspiration rates explained 52% of variation in timing of mortality in both experiments combined.

### 3.2 Variation in fitness among treatments and seed sources

Significant differences in fitness measured as survival and unconditional expected height from emergence to age 3 were detected among drought treatments in Exp. 1 (Table 2) and Exp. 2 (Table 3). Fitness was lowest in the mid‐ to late‐summer drought treatment. Fitness was highest in the late‐summer drought treatment but did not differ significantly from the mid‐ to late‐summer drought treatment (Supporting Information Figure S2). Significant variation in fitness was detected among families and for the interaction between family and treatment in Exp. 1 (Table 2). In Exp. 2, significant variation in fitness was detected among provenances; interactions between traits and provenance and between traits and treatments were also significant (Table 3). Accordingly, further assessment of phenotypic selection was based on separate analyses of treatments with family treated as a random factor. In Exp. 2, provenances were analyzed separately.

**Table 2:** Experiment 1, results from aster model comparison testing for the effects of drought treatment (TRT), maternal family (FAM), date of seedling emergence (ED), mean family seed density mg/ml (SD), early growing‐season apical shoot elongation rate (rE), and late‐season apical shoot elongation rate (rL) on survival to 2011 and seedling unconditional expected height in 2011 that included survival beginning with emergence in 2009 or alive in 2010

Response	Terms	Model *df*	Model deviance	Test *df*	Test *p*‐value	Random effects: √ of variance components (*p*‐values are one‐tailed)			
							Estimate	*SE*	*p* value
Survival									
2009–2011	TRT + FAM	40	17,760	37	<0.0001***	Block	0.282	0.0933	0.0013**
	Drought Treatment (TRT)	38	17,551	2	<0.0001***				
	Maternal Family (FAM)	5	17,675	35	<0.0001***				
	TRT:FAM	110	17,871	70	<0.0014**				
	Null	3	17,467						
	TRT + ED + SD	7	17,943	4	<0.0001***	Block	0.718	0.2352	0.0011**
	Emergence Date (ED)	6	17,702	1	<0.0001***				
	Mean Seed Density (SD)	6	17,924	1	<0.0001***				
	TRT:ED	9	17,979	2	<0.0001***				
	TRT:SD	9	17,943	2	0.7071				
	Null	3	17,467						
2010–2011	TRT + FAM	39	27,817	37	<0.0001***	Block	1.345	0.4337	0.0010**
	Drought Treatment (TRT)	39	27,758	2	<0.0001***				
	Maternal Family (FAM)	39	27,759	35	0.0076**				
	TRT:FAM	109	27,928	70	0.0014**				
	Null	2	27,696						
	TRT + rE + rL + SD	7	28,141	5	<0.0001***	Block	1.342	0.4329	0.0010**
	rE	6	28,071	1	<0.0001***				
	rL	6	27,993	1	<0.0001***				
	Mean Seed Density (SD)	6	28,138	1	0.0810				
	TRT:rE	9	28,158	2	0.0002***				
	TRT:rL	9	28,182	2	<0.0001***				
	Null	2	27,703						
Unconditional expected height									
2009–2011	TRT + FAM	40	17,944	37	<0.0001***	Block	0.001	0.0005	0.0010**
	Drought Treatment (TRT)	38	17,769	2	<0.0001***				
	Maternal Family (FAM)	5	17,811	35	<0.0001***				
	TRT:FAM	110	18,067	70	<0.0014**				
	Null	3	17,467						
	TRT + ED + SD	7	18,115	4	<0.0001***	Block	0.001	0.0004	0.0013**
	Emergence Date (ED)	6	17,832	1	<0.0001***				
	Mean Seed Density (SD)	6	18,103	1	0.0006***				
	TRT:ED	9	18,133	2	0.0002***				
	TRT:SD	9	18,117	2	0.4523				
	Null	3	17,635						
2010–2011	TRT + FAM	39	28,046	37	<0.0001***	Block	1.345	0.4337	0.0009**
	Drought Treatment (TRT)	39	28,040	2	0.0710				
	Maternal Family (FAM)	39	27,834	35	<0.0001***				
	Null	2	27,830						

*Notes*

The first model in each subsection was tested against the null model that excluded all listed terms in the first model. Individual terms were tested against the first model in each subsection. Random effects are from first model in each subsection. Row‐plot effects were negligible (NA) for all models. Significance levels: **p < .01, ***p < .001.

**Table 3:** Experiment 2, results from aster model comparison testing for the effects of timing of drought treatment (TRT), provenance (PROV) date of seedling emergence (ED), and mean family seed density mg/ml (SD), on survival to 2011 and seedling unconditional expected height in 2011 that included survival beginning with emergence in 2009 or alive in 2010

Response	Terms	Model *df*	Model deviance	Test *df*	Test *p*‐value	Random effects: √ of variance components (*p*‐values are one‐tailed)			
							Estimate	*SE*	*p* value
Survival									
2009–2011	TRT + PROV +ED + SD	9	5,766.1	6	<0.0001***	Block	0.373	0.1253	0.0015**
	Drought Treatment (TRT)	7	5,680.4	2	<0.0001***				
	Provenance (PROV)	7	5,756.9	2	0.0103*				
	Emergence Date (ED)	8	5750.1	1	<0.0001***				
	Mean Seed Density (SD)	8	5,765.6	1	0.5001				
	PROV:ED	11	5,772.3	4	0.0441*				
	TRT:ED	11	5,807.2	4	<0.0001***				
	Null	3	5,652.8						
2010–2011	TRT + PROV	7	10,368	4	0.0140*	Block	1.163	0.392	0.0015**
	Drought Treatment (TRT)	5	10,355	2	0.0014**				
	Provenance (PROV)	7	10,367	3	0.7497				
	Null	2	10,354						
Unconditional expected height									
2009–2011	TRT + PROV +ED + SD	9	5,882.2	6	<0.0001***	Block	0.002	0.0006	0.0011**
	Drought Treatment (TRT)	7	5,807.0	2	<0.0001***				
	Provenance (PROV)	7	5,867.4	2	0.0006***				
	Emergence Date (ED)	8	5,862.6	1	<0.0001***				
	Mean Seed Density (SD)	8	5,870.6	1	0.0006***				
	PROV:ED	11	5,812.9	2	0.1570				
	TRT:ED	11	5,882.2	2	<0.0001***				
	PROV:SD	11	5,888.4	2	0.0454*				
	TRT:SD	11	5,882.5	2	0.8642				
	Null	3	5,742.0						
2010–2011	TRT + PROV	7	10,545	5	<0.0001***	Block	0.004	0.001238	0.0001***
	Drought Treatment (TRT)	4	10,435	3	0.0240*				
	Provenance (PROV)	5	10,538	2	<0.0001***				
	TRT:PROV	13	10,456	6	0.3892				
	Null	2	10,491						

*Notes*

The first model in each subsection was tested against the null model that excluded all listed terms in the first model. Individual terms were tested against the first model in each subsection. Random effects are from first model in each subsection. Row‐plot effects were negligible (NA) for all models. Significance levels: *p < .05, **p < .01, ***p < .001.

### 3.3 Trait variation and correlations

Family mean seed density varied from 5.6 to 12.4 mg/ml in Exp. 1 and 8 to 11.5 mg/ml for 3 Mile, 10.4 to 11.4 mg/ml for Priest River, and 10.1 to 11.2 for Indian Prairie in Exp. 2. Seedlings emerged over a 40‐day period beginning Julian date (JD) 100 (April 10, 2009). A single seedling emerged the following year but was not included in this analysis. In Exp. 1, mean emergence date was JD 114 (*SD* = 6.32, *SE* = 0.1, *n* = 3892). In Exp. 2, mean emergence date differed significantly among provenances (*p* < 0.0001). Seeds originating from increasingly drier origins emerged earlier. In particular, mean emergence occurred on JD 114 (*SD* = 5.89, *SE* = 0.26, *n* = 498), 115 (*SD* = 6.45, *SE* = 0.29, *n* = 498), 116 (*SD* = 6.22, *SE* = 0.28, *n* = 478), for Indian Prairie, 3 Mile, and Priest River provenances, respectively. In addition, timing of seedling emergence differed significantly among maternal families within provenance (*p* < 0.0001) in Exp. 1 and Exp. 2.

Statistically significant correlations (*p* < 0.05) among traits of survivors in 2011 were detected in Exp. 1 (Supporting Information Table S1) and Exp. 2 (Supporting Information Table S2) and varied by experiment, treatment, and provenance. Correlations were lowest among traits in the late‐summer drought treatment. Emergence date and seed density were significantly correlated with growth traits in Exp. 1 but varied by treatment and seed source in Exp. 2. Early‐season elongation rates were significantly correlated with most growth traits, but only weakly and negatively correlated with late growing‐season elongation rates. Thus, phenotypes that elongated more rapidly early in the season exhibited only a slight tendency to elongate relatively slower late in the season.

### 3.4 Selection on emergence date

In both experiments, linear and quadratic terms for emergence date were significant predictors of both fitness measures, survival to 2011 beginning with emergence in 2009 and unconditional expected height in 2011 (accounting for mortality since emergence in 2009) (Supporting Information Table S3 and S4). However, the statistical significance (*p* < 0.05) of these terms differed among provenance by treatment in Exp. 2. Notably, for survival, no statistically significant (*p* < 0.05) relationship with emergence date was detected for the driest provenance, Indian Prairie, under drought imposed treatments (mid‐ to late‐summer drought and late‐summer drought treatments) or for the wettest provenance, Priest River, in the no drought treatment (Supporting Information Table S4). In addition, for unconditional expected height, no statistically significant (*p* < 0.05) relationship was detected with any predictor term for the Indian Prairie provenance in the mid‐ to late‐summer drought treatment (Supporting Information Table S4). Thus, selection was not detected in any of these specific cases.

In Exp. 1, fitness surfaces, modeled using the statistically significant (*p* < 0.05) linear and quadratic terms (see Supporting Information Table S3) and plotted in relation to observed emergence date, showed an optimum for emergence date, with phenotypes on both sides of the optimum in both the mid‐ to late‐summer and late‐summer drought treatment, thus stabilizing selection favoring intermediate emergence dates was observed most prominently in these treatments (Figure 3a,b,e). Survival and unconditional expected height declined for the many individuals having emergence date beyond the optimum, thus directional selection favoring earlier emergence accompanied stabilizing selection (Figure 3). The severity of fitness decline or magnitude of selection differed by drought treatment and between fitness measures. For fitness assessed using survival, selection against late emergence was similarly severe in both the mid‐ to late‐summer and no drought treatments and least in the late‐summer drought treatment (Figure 3). For fitness assessed using unconditional expected height, the selection against late emergence was most severe in the no drought treatment (Figure 3).

**Figure 4:**
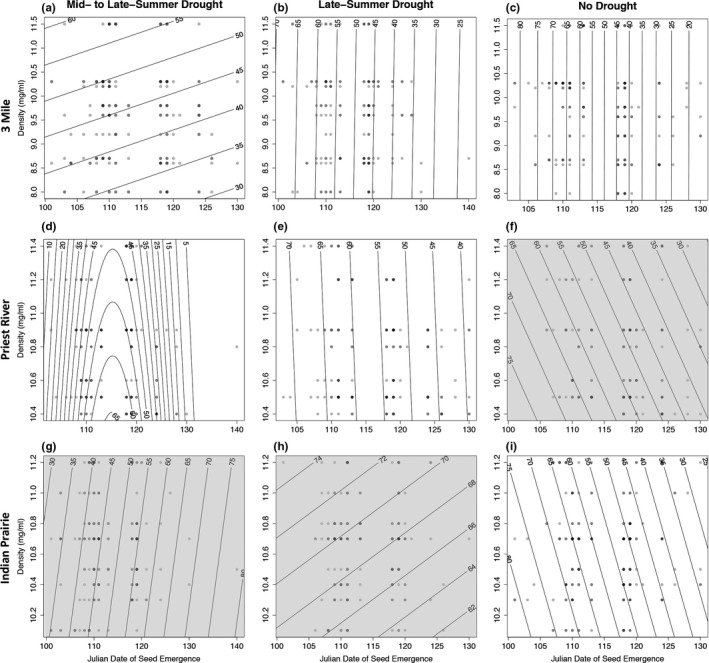
Experiment 2, fitness surfaces showing observed (circles) Julian date of emergence and mean density of seed (mg/ml) by family in relation to modeled (contour lines) survival (%) from 2009 to 2011 in the mid‐ and late‐summer (plots a, d, g), late‐summer (plots b, e, h), and no drought (plots c, f, i) treatment for seed origin from 3 Mile, Idaho (plots a,b,c), Priest River Experimental Forest, Idaho (plots d,e,f), and Indian Prairie, Montana (plots g,h,i). Increasing darkness of circles indicates higher amount of observations for the indicated measures. Density was not statistically significant (*p* < 0.05) in any plot and shows only trends (see Supporting Information Table S4). Shaded plots (f, g and h) indicate where date of emergence was also not statistically significant (*p* < 0.05)

**Figure 5:**
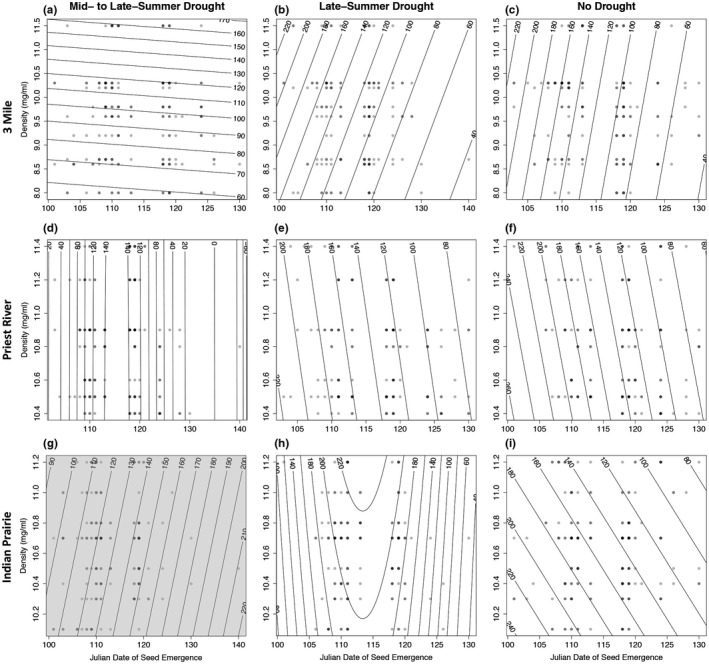
Experiment 2, fitness surfaces showing observed (circles) Julian data of emergence and mean density of seed (mg/ml) by family in relation to modeled (contour lines) unconditional expected height (mm) in 2011 that included survival beginning in 2009 in the mid‐ to late‐summer (plots a, d, g), late‐summer (plots b, e, h), and no‐drought (plots c, f, i) treatment for seed origin from 3 Mile, Idaho (plots a, b, c), Priest River Experimental Forest, Idaho (plots d, e, f), and Indian Prairie, Montana (plots g, h, i). Increasing darkness of circles indicates higher amount of observations for the indicated measures. Density but not date of emergence was statistically significant in plot (a). Neither density nor date of emergence was statistically significant (*p* < 0.05) in plot (g) (shaded plot). Date of emergence but not density was statistically significant (*p* < 0.05) in all other plots (see Supporting Information Table S4)

In Exp. 2, fitness surfaces for survival plotted in relation to mean seed density and emergence date indicated a tendency toward directional selection favoring increasingly early emergence dates (Figure 4a,b,c,e,i), with a single case of stabilizing selection favoring an intermediate emergence date (Figure 4d). Fitness surfaces based on unconditional expected height also generally showed directional selection favoring increasingly earlier emergence dates (Figure 5b,c,e,i) and increasing seed density (Figure 5a). For the Indian Prairie provenance in the late‐summer drought treatment stabilizing selection favoring an intermediate date was observed (Figure 5h).

### 3.5 Selection on family mean seed density

Fitness regarded as either survival alone beginning in 2009 in Exp. 1 or unconditional expected height in 2011 (which, again, accounts for mortality since 2009) in Exp. 1 and 2 depended on family mean seed density (mass per unit volume, Tables 2 and 3). In Exp. 2, the interaction between seed density and treatment significantly influenced unconditional expected height (*p* < 0.05). Fitness surfaces for survival plotted in relation to seed density show directional selection favoring increasing seed density (Figures 3 and 5). When the aster analysis included only those surviving to 2010, no significant influence on either survival or unconditional expected height in 2011 was detected. Thus, selection on mean seed density predominantly resulted from mortality in the first year.

### 3.6 Selection on timing and magnitude of shoot elongation

**Figure 6:**
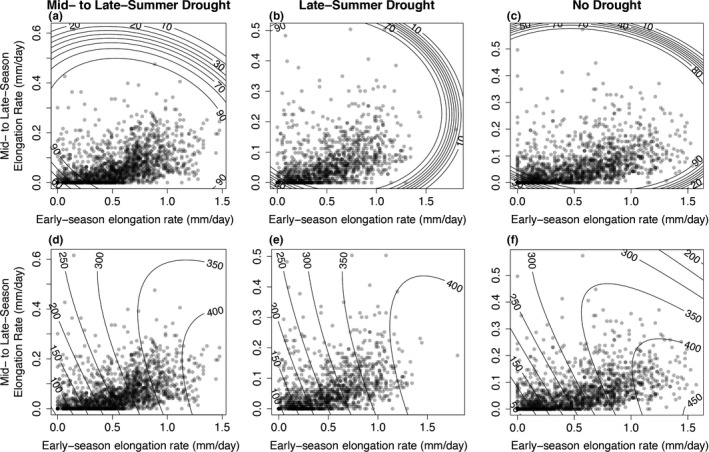
Experiment 1, fitness surfaces showing observed (circles) apical shoot elongation rates (mm/day) early (March 6 to May 7) and mid‐ to late season (May 8–Sept 28) in 2010 in relation to modeled (contour lines) survival (a, b, and c) and unconditional expected height (d, e and f) in 2011 that included survival beginning in 2010 in the mid‐ to late‐summer, late‐summer, and no‐drought treatment. Increasing darkness of circles indicates higher amount of observations for the indicated measures

Fitness regarded as either survival alone beginning in 2010 or unconditional expected height in 2011 that accounts for mortality beginning in 2010 depended on the rate of apical shoot elongation in 2010 (Table 2, Supporting Information Table S5). In Exp. 1, linear and quadratic terms and the cross‐products for the timing of shoot elongation were significant predictors of fitness (Supporting Information Table S5). Plotted observations of shoot elongation on fitness surfaces based on survival alone showed selection predominantly against the slowest early‐ and late‐season shoot elongation. The magnitude of selection was greatest in the no drought treatment (Figure 6c) and least in the mid‐ to late‐summer drought treatment (Figure 6a). The shapes of the fitness surfaces for unconditional expected height were generally consistent across all drought treatments (Figure 6d,e,f) showing selection favoring phenotypes with increasingly higher early‐season growth rates. In addition, selection slightly favored lower mid‐ to late‐season growth rates. In Exp. 2, linear and quadratic terms for the timing of shoot elongation were significant predictors of fitness (Supporting Information Table S6). Selection favored increasing early‐season shoot elongation (Supporting Information Figure S3). The interactions between treatment and provenance were not significant either for survival or unconditional expected height (Table 3). Thus, differences in selection on growth rates among provenances were not detected.

## 4 DISCUSSION

Evolutionary response is expected when selection acts on a trait that varies heritably within a population (Darwin, 1859; Endler, 1986; Falconer & Mackay, 1996). Results from the present study demonstrate and characterize phenotypic selection on traits that have been shown to be heritable and variation in selection among populations from three climatically distinct seed origins under experimentally imposed drought treatments in the field.

At the extremely dry experimental site, treatments were imposed in the year of seedling emergence by eliminating weekly irrigation in late June (mid‐ to late‐summer drought) or late July (i.e., late‐season drought), or by continuing irrigation through the growing season (no drought). No further irrigation was applied in subsequent years. Selection was generally more acute on populations for which patterns of precipitation at the site of seed origin differed most from the drought treatment. For example, for the seed source with the lowest annual precipitation, Indian Prairie (Table 1), selection was detected in the no drought treatment but not in the mid‐ to late‐summer drought treatment (Supporting Information Table S4, Figures 4g,h, and 5g). In addition, for the seed source with the highest annual precipitation, Priest River (Table 1), selection was detected in the drought‐induced treatments but not in the no drought treatment (Supporting Information Table S4, Figure 4f). These results align with studies that have identified clinal trait variation in relation to moisture stress of seed origin (e.g., Ager & Stettler, 1983; Kolb, Grady, Mcettrick, & Herrero, 2016; Moore & Kidd, 1982; Rehfeldt, 1991; Weber & Sorensen, 1992) and support an interpretation that populations of ponderosa pine are differentially adapted to drought patterns at their geographic origin. Accordingly, the evolutionary response to future droughts would be expected to differ among populations.

The present study assessed natural selection during seedling emergence and early growth using seedlings that overwintered as seed germinated and grew entirely in the field. Therefore, these results are expected to more closely reflect selection under conditions that approximate natural regeneration in comparison with studies that use seedlings grown in growth chambers or greenhouse conditions or for seedling outplanted into the field (see Poorter et al., 2016). In particular, root systems of seedlings grown from seed at the study site were unimpeded by pots or artificial boundaries (see Halter & Chanway, 1993; Halter, Chanway & Harper, 1993; Young & Evans, 2000). This is important because initial root development is critical for establishing access of seedlings to soil moisture and, hence, for their survival through drought (Bates, 1923). The taproot of ponderosa pine can penetrate soil to an average of 58 cm in the first growing season (Larson, 1963). Thus, the use of alternative methods such as raised beds or containers would likely obscure natural selection associated with variation in root development.

Fitness is generally defined as the number of offspring contributed to the next generation and is consequently especially difficult to measure for long‐lived trees. Most studies that assess fitness for tree species use measures of survival as a partial measure or height as a proxy. Survival is a fundamental component of fitness, while relative height can confer fitness by, for example, mediating access to resources (King, 1990) and allowing for greater reproductive surface (see McGraw & Wulff, 1983). Accordingly, we assessed fitness using survival and unconditional expected height to represent fitness to provide a more complete and informative analysis of selection in comparison with analyses that used either survival or height alone (see Rousi, Possen, Ruotsalainen, Silfver, & Mikola, 2018; Savolainen et al., 2007; Warwell & Shaw, 2017). For long‐lived organisms, fitness is often evaluated over short periods. Here, the analyses of fitness span from the earliest life stages through age 3.

Selection analysis using a fitness regression approach can estimate selection on a trait but cannot definitively confirm that a trait is under direct selection without meeting the unlikely condition that all correlated traits under selection are included in the analysis (Lande & Arnold, 1983). Nonetheless, insight into possible mechanisms of selection can be gained through analyses that include a carefully considered range of traits with regard to their function (Mitchell‐Olds & Shaw, 1987).

Early emergence date has been positively associated with increased growth and survival in ponderosa pine grown under nursery conditions (Mexal & Fisher, 1987), in Scots pine (*P. sylvestris*) grown in experimental drought treatments imposed across three microhabitats in natural mountain forests in southeast Spain (Castro, 2006), and in a wide variety of plant life forms over a broad range of environments (see Verdu & Traveset, 2005). Hypotheses explaining this apparently common relationship have been grouped into two broad categories that ascribe it to either environmental effects or maternal/genetic effects (Jones, Allen, & Sharitz, 1997). Environmental explanations attribute increased growth and survival of earlier emergents to greater access to ephemerally available resources such as water or nutrients (Jones & Sharitz, 1989). In contrast, maternal/genetic explanations attribute greater growth and survival among earlier emergence to adaptive genotypes or traits such as seedling vigor which may be manifested in early emergence (Dunlap & Barnett, 1983; McDaniel, 1969).

In the present study, growing conditions were more favorable in spring and early summer. The environmental hypothesis would predict higher fitness in association with earlier spring dates of emergence particularly when drought occurred early in the summer. Higher fitness was associated with generally early emergence, but with an intermediate optimum in drought imposed treatments, although not in the no drought treatment (Figure 3). Thus, results do not support a purely environmental explanation and instead suggest that selection on date of emergence was also influenced by genetic/maternal factors (see family effects, Table 2).

The present study also detected a positive trend for earlier emergence in association with increasingly drier seed origins which suggests that timing of seed emergence may vary clinally. This finding corresponds with Weber and Sorensen (1992) who presented evidence that speed of germination among central Oregon provenances varied positively with decreasing precipitation at the seed origin, as inferred from geographic variables.

Seed density (mass per unit volume) and seed weight appear to act independently although both have been shown to affect seedling vigor (*Glycine max*, Hoy & Gamble, 1985). Results in the present study identify directional selection favoring increasing mean seed density in both experiments that was more pronounced with increased moisture stress (Tables 2 and 3, Figures 3 and 5). These results agree with previous studies that identify a positive relationship between seed density and plant performance under moisture stress environments (e.g., Hoy & Gamble, 1985; *Pennisetum americanum*, Gardner & Vanderlip, 1989). Given that seed density is positively associated with seedling vigor (*Gossypium hirsutum*, Krieg & Bartee, 1975; McDonald, 1975; Hoy & Gamble, 1985) and selection on seed density was only detected in association with mortality in the 1st year (Tables 2 and 3), results from the present study suggest that observed selection favoring increasing seed density may have resulted from direct selection favoring increasing seedling vigor under increasing moisture limiting conditions in the growing season of emergence.

Growth rhythm represents the timing of an intercorrelated suite of annual growth processes in relation to annual climatic events (cf. Dietrichson, 1964; Howe et al., 2003; Rehfeldt, 1992). The synchronization of growth rhythm with local climate is a key component of adaptation in many forest tree species (Morgenstern, 1996). Growth rhythm encompasses the timing and rates of shoot elongation; selection on these traits suggests selection on many other components of growth rhythm, such as date of bud set and timing of dormancy as well. Indeed, in the present study, early‐season elongation rate was well correlated with most measured traits (Supporting Information Tables S1 and S2). Notably, directional selection favored increasing early‐season shoot elongation rates in all treatments in both experiments. However, the analysis of selection on shoot elongation considered only those trees surviving at the end of the first year, after 96% of total mortality over the 3‐year study period occurred, because this trait was not expressed for the first time until 2010. Therefore, the shoot elongation rates of seedlings that died in 2009 could not be accounted for or jointly assessed in relation to selection on emergence dates. Selection on growth rhythm could also occur through this “invisible fraction” (see Bennington & McGraw, 1995). Further investigation may benefit from the general approach used by Mojica and Kelly (2010), who assessed the effect of selection on the invisible fraction using individuals with independently established genotype and phenotypic expression.

Over the 3‐year study period, we detected a commonality of higher fitness in the late‐summer drought treatment in comparison to the mid‐ to late‐summer drought treatment and no drought treatment (Supporting Information Figure S2). This result likely reflects a general adaptation to late‐summer drought of populations in the study region, which is characterized by late‐summer drought. Thus, climate change that alters precipitation through the growing season may reduce fitness.

Findings from this study are particularly relevant to resource managers given that climate for western North America is expected to become warmer and drier with an increase in the temporal frequency and spatial extent of growing‐season drought events through the 21st century (Sheffield & Wood, 2008). In particular, the findings can assist managers in early identification of better performing phenotypes for warm‐dry climate characterized by droughts. Results showed that phenotypes with high seed density, an intermediate but relatively early emergence date and high 2nd‐year early‐season shoot elongation exhibited the greatest estimated fitness after three years in the field under a climate warmer and drier than seed origins. The detection of higher mean fitness among seed source originating from driest origin in the drier tests suggests that populations originating from drier origins may have more progeny that are adapted to drought. This result agrees with Rehfeldt (1992) who identified higher early‐season growth rates for the drier Indian prairie seed source in comparison with 3 Mile seed sources. *Ex situ* gene conservation operations should consider including seed originating from the driest locations within a region. Nevertheless, the detection of phenotypic selection on seed source that originated from wetter origins in the drier treatments indicates the operation of selection that could adapt such populations to warmer‐drier conditions in the immediate future.

Selection was assessed in the first 3 years following emergence. However, selection due to differential survival and growth following age three is likely common (see Bakker, Moore, & Covington, 2006; Warwell & Shaw, 2018) and changes in early relative height among families that are thought to be linked to changes in competitive interactions have been observed in ponderosa pine between age 20 and 25 years (Namkoong & Conkle, 1976). Therefore, projection of fitness beyond the three‐year‐old span assessed in the present study represents an extrapolation that demands caution. Furthermore, the present results were conducted in only one location, and because biotic interactions were controlled, their effects were not assessed. Therefore, natural selection on the traits examined under wild conditions could differ substantially even under similar climate conditions.

Selection via differential mortality among families in the year of emergence was particularly strong, while selection in subsequent years was far less pronounced. Therefore, natural selection in the year of seed emergence may represent an important stage for drought adaptation among forest tree populations. Maternal effects, which are effects on a progeny's phenotype that originate from the maternal parent during seed development, could not practically be assessed or eliminated. Consequently, direct genetic and maternal effects are confounded in the present study. Therefore, to the extent that the traits examined are heritable or correlated with heritable traits, results suggest that natural selection specific to drought conditions in the year of emergence may substantially influence climate adaptation. Certainly, the role of selection in the year of emergence should be further investigated, particularly given that reforestation practices likely alter selection during this period by growing seedlings under nursery conditions and planting out survivors in later years.

## CONFLICT OF INTEREST

None declared.

## DATA ARCHIVING STATEMENT

Data will be available from the USDA, Forest Service Research Data Archive: https://www.fs.usda.gov/rds/archive/ one year following the date of publication.
