# Supplementary material for: Phenotypic selection on ponderosa pine seed and seedling traits in the field under three experimentally manipulated drought treatments
Source: Evol Appl. 2018 Dec 19;12(2):159–74. doi: 10.1111/eva.12685 (PMC6346659; doi:10.1111/eva.12685)
Supplement: Supplementary file 1 [file EVA-12-159-s001.docx]

Table S1. Experiment 1, Spearman’s rank correlations for family mean seed density (Density), emergence date in the 1^st^ year ( E Date), rate of shoot elongation during March 6 – May 7: early-growing season: (rE), May 8-June 2: mid-growing season (rM), June 3 - Sept 28: late-growing season (rR), and the total growing season (Total Rate) in 2010, apical bud length and lammas growth in fall 2010 (Bud10) and 2011 (Bud11) in the Mid- to Late-Summer (n=335), Late-Summer (n=490), and No (n=437) drought treatments. Correlations consider only live trees in 2011.

Note: ***** P < .05, ****** P < .01, ******* P < .001, a = This column shows correlations that used family means for each trait combination.

Table S2A. Experiment 2, 3 Miles, Idaho provenance, Spearman’s rank correlations for maternal family mean seed density (Density), emergence date in the 1^st^ year ( E Date), rate of shoot elongation during March 6 – May 7: early-growing season: (rE), May 8-June 2: mid-growing season (rM), June 3 - Sept 28: late-growing season (rL), and the total growing season (Total Rate) in 2010, apical bud length and lammas growth in fall 2010 (Bud10) and 2011 (Bud11) in the Mid- to Late-Summer (n=36), Late-Summer (n=44) and No (n=43) drought treatments. Correlations consider only live trees in 2011.

Note: ***** P < .05, ****** P < .01, ******* P < .001, a = This column shows correlations that used family means for each trait combination.

Table S2B. Experiment 2, Priest River, Idaho provenance, Spearman’s rank correlations for maternal family mean seed density (Density), emergence date in the 1^st^ year ( E Date), rate of shoot elongation during March 6 – May 7: early-growing season: (rE), May 8-June 2: mid-growing season (rM), June 3 - Sept 28: late-growing season (rL), and the total growing season (Total Rate) in 2010, apical bud length and lammas growth in fall 2010 (Bud10) and 2011 (Bud11) in the Mid- to Late-Summer (n=38) Late-Summer (n=45) and No (n=47) drought treatments. Correlations consider only live trees in 2011.

Note: ***** P < .05, ****** P < .01, ******* P < .001, a = This column shows correlations that used family means for each trait combination.

Table S2C. Experiment 2, Indian Prairie, Montana provenance, Spearman’s rank correlations for maternal b family mean seed density (Density), emergence date in the 1^st^ year ( E Date), rate of shoot elongation during March 6 – May 7: early-growing season: (rE), May 8-June 2: mid-growing season (rM), June 3 - Sept 28: late-growing season (rL), and the total growing season (Total Rate) in 2010, apical bud length and lammas growth in fall 2010 (Bud10) and 2011 (Bud11) in the Mid- to Late-Summer (n=41), Late-Summer (n=57), and No (n=55) drought treatments. Correlations consider only live trees in 2011.

Note: ***** P < .05, ****** P < .01, ******* P < .001, a = This column shows correlations that used family means for each trait combination.

Table S3. Experiment 1, results from aster model comparison testing for the effects of mean seed density (mg/ml), emergence date and its quadratic on survival though 2011 beginning in 2009 and 2010 and unconditional expected height (mm) in 2011 that included survival beginning in 2009 and 2010.

β= linear selection gradient, γ_ii_ = quadratic effects , Full models included all presented variables listed directly under the term, Random effects are from models using emergence date and its quadratic or null model with no fixed effects in treatments where emergence date was not significant. Estimated selection gradients (partial regression coefficients) and standard errors represent results from models that used only seed density or only date of emergence. Row-plot effects were negligible (NA) for all models.

Table S4. Experiment 2, results from aster model comparison testing by provenance for the effects of mean seed density (mg/ml), emergence date and its quadratic on survival though 2011 beginning in 2009 and unconditional expected height (mm) in 2011 that included survival beginning with emergence in 2009.

Table S4. Continued

β= linear selection gradient, Full models included all presented variables listed directly under the term. Null models exclude all presented variables listed above the term. The effect of each predictor variable was tested against the full model. Random effects are from model using density term and significant terms for emergence date. Row-plot effects were negligible (NA) for all models

Table S5. Experiment 1, results from aster model comparison testing the effects of mean seed density (mg/ml), early- and late-season shoot elongation rates (mm) in 2010 and their quadratic and cross products on unconditional expected height (mm) in 2011 that included survival from 2010 through 2011.

β= linear selection gradient, γ_ii_ = quadratic effects, γ_ij =_ cross products, Full models included all presented variables listed directly under the term. The effect of each predictor variable was tested against the full model for each predictor variable. Selection gradients, standard error and random effects are from models that include only significant terms. Row-plot effects were negligible (NA) for all models

Table S6. Experiment 2, results from aster model comparison testing the effects of mean seed density (mg/ml), early- and late-season shoot elongation rates (mm) in 2010 and their quadratic and cross products on unconditional expected height (mm) in 2011 that included survival from 2010 through 2011.

Table S6. Continued

β= linear selection gradient, γ_ii_ = quadratic effects, γ_ij =_ cross products, Full models included all presented variables listed directly under the term. The effect of each predictor variable was tested against the full model for each predictor variable. Selection gradients, standard error and random effects are from models that include only significant terms. Row-plot effects were negligible (NA) for all models


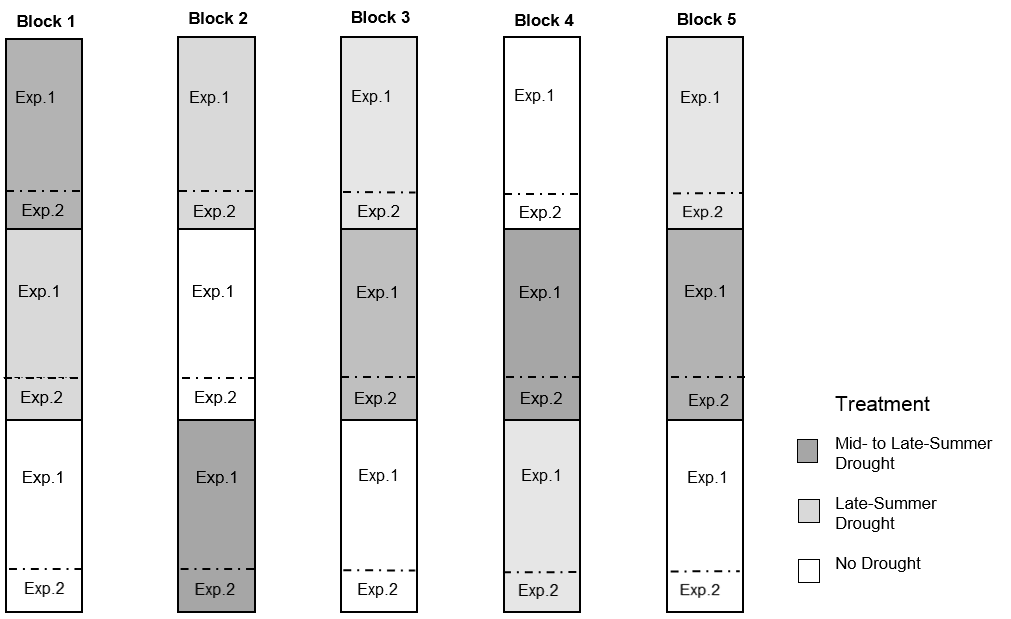


Figure S1. Study design of field planting showing Experiment 1 (Exp.1) and Experiment 2 (Exp. 2) interplant in a randomized complete block design, with five blocks and three drought treatments randomized within each block.


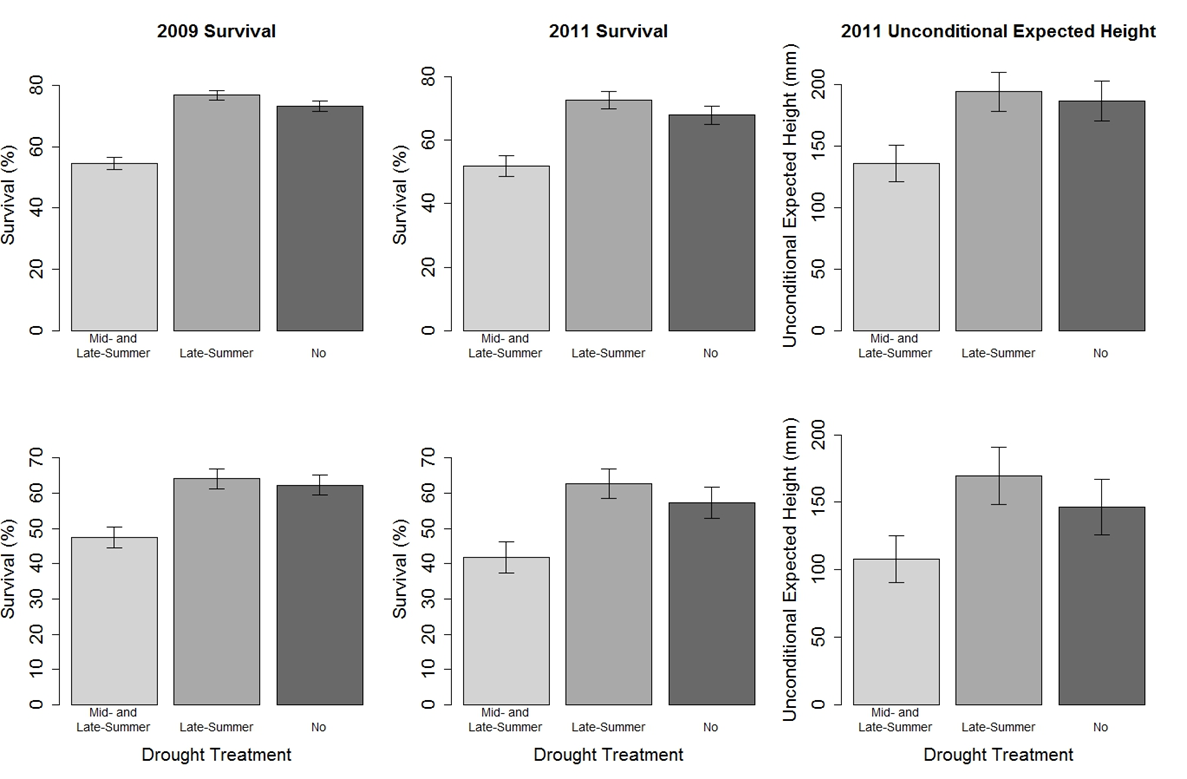


Figure S2. Modeled survival (%) (±1SE) in 2009 and 2011and unconditional expected height (mm) (±1SE) in 201l by Mid- and Late-Summer, Late-Summer and No drought treatment in Experiment 1 (top row) and Experiment 2 (bottom row).


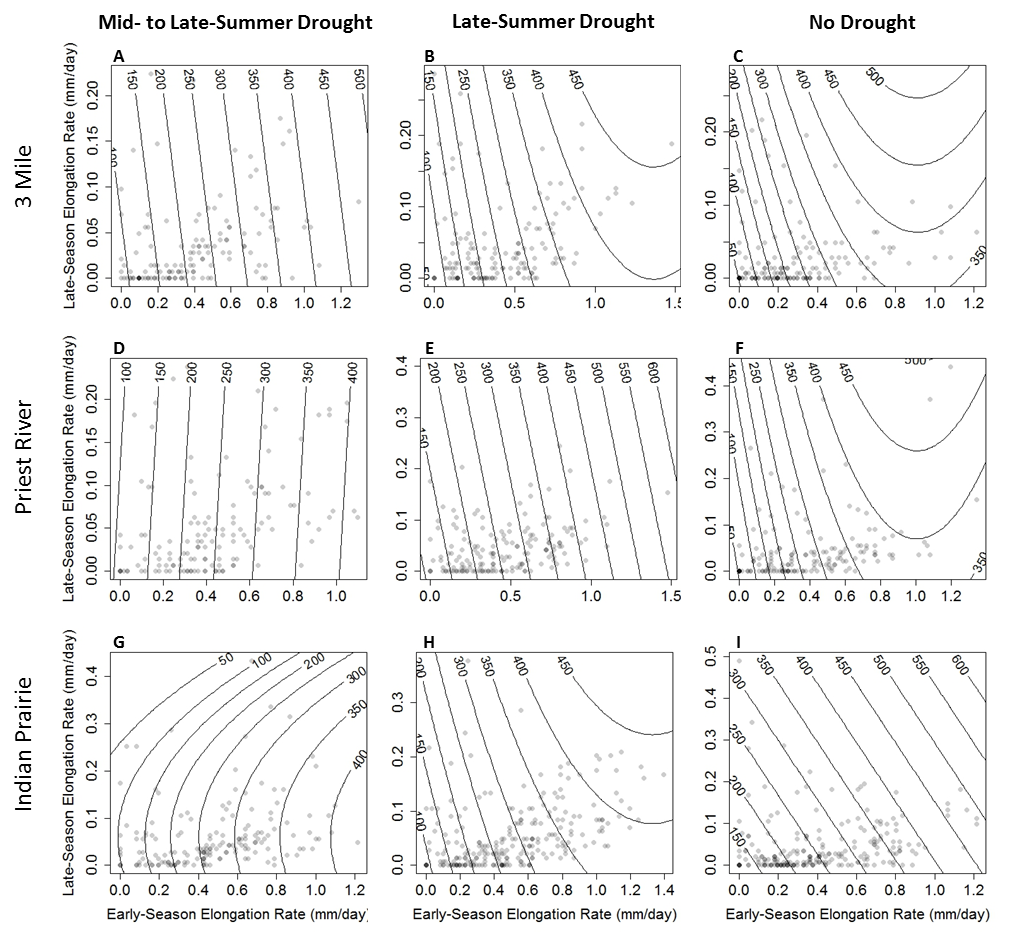


Figure S3. Experiment 2, fitness surfaces showing observed (circles) apical shoot elongation rates (mm/day) early (March 6 to May 7) and mid- to late-season (May 8 –Sept 28) in 2010 in relation to modeled (contour lines) unconditional expected height in 2011 that included survival beginning in 2009 in the Mid- to Late-Summer, Late-Summer and No drought treatment for seed source from 3 Mile, Idaho, Priest River Experimental Forest, Idaho and Indian Prairie, Montana. Increasing darkness of circles indicates higher amount of observations for the indicated measures.
